# Indoleamine-2,3-Dioxygenase 1 Deficiency Suppresses Seizures in Epilepsy

**DOI:** 10.3389/fncel.2021.638854

**Published:** 2021-02-18

**Authors:** Ning Deng, Jiao Hu, Yu Hong, Yuewen Ding, Yifan Xiong, Zhiyong Wu, Wei Xie

**Affiliations:** ^1^School of Traditional Chinese Medicine, Southern Medical University, Guangzhou, China; ^2^Department of Traditional Chinese Medicine, Nanfang Hospital, Southern Medical University, Guangzhou, China

**Keywords:** IDO1, epilepsy, kynurenine, tryptophan, inflammation, oxidative stress

## Abstract

**Background**: Indoleamine-2,3-dioxygenase 1 (IDO1) is the initial and rate-limiting enzyme in the metabolism of tryptophan (TRP) to kynurenine (KYN). IDO1-dependent neurotoxic KYN metabolism plays a crucial role in the pathogenesis of many neurodegenerative disorders. However, the function of IDO1 in epilepsy is still unclear.

**Objective**: In this study, we investigated whether IDO1 deficiency could affect epilepsy in a lithium-pilocarpine-induced model.

**Methods**: Patients with epilepsy and controls were enrolled. Male C57BL/6 mice and IDO1 knockout (KO, IDO1^−/−^) mice were subjected to intraperitoneal injection of lithium and pilocarpine to induce epilepsy. The levels of IDO1 and concentrations of TRP and KYN in patients with epilepsy and epileptic mice were evaluated by enzyme-linked immunosorbent assay (ELISA) and liquid chromatography-mass spectrometry (LC-MS), respectively. Then, behavioral phenotypes related to epileptic seizures and neuronal damage were compared between KO and wild-type (WT) mice with lithium-pilocarpine-induced epilepsy. To explore the underlying pathways involved in the effects of IDO1 deficiency, the concentrations of kynurenic acid (KYNA) and quinolinic acid (QUIN), glial cell activation, the levels of major pro-inflammatory cytokines, and antioxidant enzyme activity were measured by LC-MS, immunohistochemistry, and ELISA.

**Results**: In this study, IDO1 levels and the KYN/TRP ratio in the sera and cerebrospinal fluid (CSF) were increased in patients with epilepsy. Also, IDO1 levels, the KYN/TRP ratio, and the levels of pro-inflammatory cytokines in the sera and hippocampi were increased in mice during the acute phase and chronic phase after status epilepticus (SE). Furthermore, IDO1 was localized in microglial cells in epileptic mice. IDO1 deficiency delayed SE onset and attenuated the frequency, duration, and severity of spontaneous recurrent seizures (SRSs). Moreover, IDO1 deficiency improved neuronal survival. Additionally, IDO1^−/−^ epileptic mice showed progressive declines in QUIN production, glial cell activation and pro-inflammatory cytokines levels, and enhanced antioxidant enzyme activity.

**Conclusions**: IDO1 deletion suppressed seizures and alleviated neuronal damage by reducing the IDO1-dependent production of neurotoxic metabolites, which finally inhibited glial cell activation and pro-inflammatory cytokine production and improved antioxidant enzyme activity. Our study demonstrates that IDO1 may be involved in the pathogenesis of epilepsy and has the potential to be a therapeutic target for epilepsy treatment.

## Introduction

Epilepsy, one of the most common chronic neurological disorders, is characterized by spontaneous and recurrent brain seizures and affects approximately 65 million people worldwide (Vezzani et al., 2019). Despite the availability of antiepileptic drugs, seizures cannot be controlled in approximately 30% of patients. Temporal lobe epilepsy is the most common type of epilepsy and is often refractory to pharmacologic treatment (Gross et al., 2017). Therefore, understanding the mechanisms that underlie the pathogenesis of epilepsy is of great importance.

Indoleamine-2,3-dioxygenase 1 (IDO1) is an intracellular enzyme that catalyzes the first step of the pathway through which tryptophan (TRP) is converted to kynurenine (KYN). KYN can be metabolized to kynurenic acid (KYNA), anthranilic acid, and 3-hydroxykynurenine (3-HK), which is further converted to quinolinic acid (QUIN; Heisler and O’Connor, 2015). QUIN, a metabolite of the KYN pathway, has a neurotoxic effect by activating *N*-methyl-D-aspartate (NMDA) receptors (Iwaoka et al., 2020). QUIN can also evoke neurotoxicity by inducing glial activation, free radical production, and oxidative stress (Fujigaki et al., 2017). KYNA is considered to play a neuroprotective role by serving as an antagonist of ionotropic glutamate receptors. However, the abnormal accumulation of KYNA can lead to inflammation (Berlinguer-Palmini et al., 2013; Małaczewska et al., 2014). Pathological activation of the KYN pathway has been implicated in a variety of neurodegenerative diseases and neuropsychiatric diseases. Increased KYN content and IDO1 activity have been detected in the blood of Huntington’s disease patients (Boros et al., 2019). It has been reported that neurons in the striatum of IDO1 knockout (KO, IDO1^−/−^) mice become less sensitive to QUIN-induced neurotoxicity (Mazarei et al., 2013). Elevated expression of IDO1, which results in the promotion of the KYN pathway, has been observed in the brain in the context of Alzheimer’s disease (Lovelace et al., 2017). Depression has been associated with dysregulation of the KYN pathway. Depressive-like behaviors are abrogated by genetic deletion or pharmacological inhibition of IDO1 (Platten et al., 2019). Our previous research demonstrated that activation of IDO1 contributes to epilepsy-associated depressive-like behavior. The IDO1 inhibitor 1-methyltryptophan alleviates depressive-like behavior but fails to relieve spontaneous seizures (Xie et al., 2014). Although 1-methyltryptophan can inhibit IDO1 enzyme activity, it lacks specificity for IDO1 (Yeung et al., 2015). One strategy for effectively and specifically inhibiting IDO1 is suppressing the IDO1 gene. Further studies involving IDO1 gene-deficient mice are needed. A recent study suggested that IDO1 deletion promotes seizures and neuropathogenesis in acute Theiler’s murine encephalitis virus (TMEV)-induced encephalitis (Juda et al., 2019). IDO1 is upregulated by inflammatory mediators, such as interleukin (IL)-1β, IL-6, tumor necrosis factor-α (TNF-α), and interferon-γ (Jiang et al., 2018). Thus, IDO1 is thought to be activated during seizures secondary to encephalitis. However, whether IDO1 is involved in the development of primary epilepsy is still unclear.

Therefore, in this study, we explored the role of IDO1 in epilepsy. First, we measured IDO1 levels and the KYN/TRP ratio in patients with epilepsy and an epileptic mouse model. Then, we utilized IDO1^−/−^ mice to assess the effect of IDO1 on seizures in a lithium-pilocarpine-induced epilepsy model. To further explore the mechanisms of IDO1 in epilepsy, we evaluated the effects of IDO1 deficiency on neuronal loss, KYN metabolite levels, the inflammatory response, and oxidative stress in epileptic mice.

## Materials and Methods

### Subjects

Twenty-one patients with epilepsy and 13 controls were recruited from the Neurology Department of Nanfang Hospital, Southern Medical University. These 21 patients were divided into the primary epilepsy group (*n* = 9) and the seizures secondary to autoimmune encephalitis group (*n* = 12) according to the cause of epilepsy. Of these 21 patients with epilepsy, 11 had status epilepticus (SE) and 10 did not. All patients were diagnosed by their supervisory doctors according to criteria established by the International League Against Epilepsy (Kwan et al., 2010; Scheffer et al., 2017). None of the control subjects had a history of epilepsy or other neurological diseases or had been exposed to antiepileptic drugs. The study was approved by the Ethics Committee of Nanfang Hospital, Southern Medical University.

### Animals

Male C57BL/6 mice (6–8 week of age) were purchased from the Experimental Animal Center of Southern Medical University (Guangzhou, China). IDO1^−/−^ mice were obtained from the Jackson Laboratory (Bar Harbor, ME, USA) and wild-type (WT) littermates were produced by heterozygous mating. The IDO1^−/−^ mouse genotyping results are presented in Supplementary Figure 1. The animals were maintained in specific pathogen-free facilities at a temperature of 22 ± 1°C on a 12 h light/12 h dark cycle and were provided free access to standard food and water. All animal procedures performed in this study were approved by the Institutional Animal Care and Use Committee of Southern Medical University (permit number: 00197090).

To study the temporal dynamics of IDO1 levels and activity in the lithium-pilocarpine-induced model of epilepsy, mice were randomly divided into seven groups according to the time at which they were evaluated after SE: the control group, 1 day group, 3 days group, 1 week group, 2 weeks group, 1 month group and 2 months group. Experimental mice were sacrificed at the corresponding time points.

To assess the effect of IDO1 deficiency on epilepsy, mice were randomly allocated into four groups: the WT control group, the WT model group, the IDO1^−/−^ control group, and the IDO1^−/−^ model group. Mice were sacrificed and subjected to ELISA, LC-MS, histopathological examination, and immunofluorescence 2 months after SE.

### SE Induction and Monitoring of Spontaneous Recurrent Seizures (SRSs)

Mice were intraperitoneally (i.p.) injected with lithium chloride (127 mg/kg, Sigma–Aldrich, St. Louis, MO, USA) and then 20 h later with pilocarpine hydrochloride (30 mg/kg, i.p., Sigma–Aldrich, St. Louis, MO, USA) to induce SE. To reduce the peripheral side effects of pilocarpine, methyl scopolamine nitrate (1 mg/kg, i.p., Tokyo Chemical Industry, Tokyo, Japan) was given 30 min before pilocarpine administration. The severity of behavioral seizures was graded according to Racine’s scale (Racine, 1972). Only mice with at least stage 3 seizures were selected for further experimentation. Diazepam (15 mg/kg, i.p., King York, Tianjin, China) was administered to terminate behavioral seizures 2 h after SE. Mice in the control group were handled similarly but received an equal volume of saline instead of lithium-pilocarpine.

The SRSs of mice were monitored by video from day 28 to day 41 after SE. Only generalized convulsive stage 4 and 5 seizures were observed (Wang et al., 2015; Lin et al., 2017; Yang et al., 2017). All video recordings were analyzed by independent investigators blinded to the study.

### Sample Preparation

#### Human Samples

All human blood samples were collected in vacuum tubes and then allowed to coagulate at 37°C for 30 min. The serum was separated by centrifugation at 3,000 rpm for 15 min. Cerebrospinal fluid (CSF) samples were collected by lumbar puncture and centrifuged at 1,000 rpm for 10 min. All serum and CSF samples were stored at −80°C until analysis.

#### Animal Samples

At the end of the experiments, mice were anesthetized with sodium pentobarbital. The procedure used to collect animal serum was the same as that used to collect human serum samples. For histopathological examination and immunofluorescence staining, mice were perfused with phosphate-buffer-saline (PBS). Then, the whole brains were removed and post-fixed in 4% paraformaldehyde. For ELISA and LC-MS analysis, the hippocampi were collected quickly and stored at −80°C.

### Biochemical Analyses

#### Analysis of Human Samples

IDO1 levels in the sera and CSF of subjects were measured by a human IDO enzyme-linked immunosorbent assay (ELISA) kit (Cusabio Biotech, Wuhan, China). ELISA was performed following the manufacturer’s instructions. The optical density was determined with a plate reader (BMG LabTech, Germany).

#### Analysis of Animal Samples

Frozen hippocampal tissues were diluted in ice-cold PBS at a concentration of 10% (w/v) after being weighed and homogenized. Then, the tissue homogenates were centrifuged at 4°C for 15 min, and the supernatants were collected for measurement. IDO1 levels in the sera and hippocampal tissue supernatants were assessed using a mouse IDO1 ELISA kit (Cusabio Biotech, Wuhan, China). The levels of inflammatory factors, such as IL-1β, IL-6, and TNF-α, in the sera and hippocampal tissue supernatants were measured by ELISA (Boshen Biotechnology, Nanjing, China). The activity of SOD, GSH-Px, and CAT and MDA content in the sera and hippocampal tissue supernatants were measured using kits (Jiancheng Bioengineering Institute, Nanjing, China) according to the manufacturer’s instructions.

### Liquid Chromatography-Mass Spectrometry (LC-MS) Analysis of TRP, KYN, QUIN and KYNA

#### Analysis of Human Samples

The concentrations of TRP and KYN in the sera and CSF of subjects were measured by the combination of high-performance liquid chromatography (LC-30AD, Shimadzu, Kyoto, Japan) and triple quadruple mass spectrometry (Triple Quad 4500, AB Sciex, Boston, MA, USA). The parameters of the mass spectrometer and the mobile phase were set as previously described (Wang et al., 2018). IDO1 activity was determined according to the KYN/TRP ratio.

#### Analysis of Animal Samples

Hippocampal tissues were thawed and then homogenized in an ice-cold extraction solution. Homogenates were centrifuged at 4°C for 10 min, and supernatants were collected for analysis. The concentrations of TRP, KYN, QUIN, and KYNA in the sera or hippocampal tissue supernatants were measured by LC-MS.

### Hematoxylin-Eosin (H&E) Staining and Nissl Staining

The whole brains of mice were post-fixed with 4% paraformaldehyde overnight at room temperature, dehydrated in a gradient alcohol series, and embedded in paraffin. Then brain tissues were sectioned into 3 μm thick slices. For H&E staining, brain slices were stained with hematoxylin and eosin. Brain slices were processed for Nissl staining using toluidine blue. The slices were observed, and random images of each sample were taken under a microscope (Olympus, Tokyo, Japan).

### Immunofluorescence Staining

Brains were post-fixed in 4% paraformaldehyde for 24 h and subsequently penetrated with 15% sucrose and 30% sucrose. Then, the brain tissues were sectioned into 6 μm thick coronal slices. The brain slices were blocked with 5% normal donkey serum and 0.1% Triton X-100 in PBS for 60 min at room temperature. For double immunofluorescence staining, the brain slices were incubated with a mixture of primary antibodies, including anti-IDO1 (rabbit polyclonal, 1:50; Proteintech, Rosemont, IL, USA), anti-GFAP (goat polyclonal, 1:200 Abcam, Burlingame, CA, USA), and anti-Iba1 (goat polyclonal, 1:100, Novus Biologicals, Centennial, CO, USA). The brain slices were then incubated with a mixture of Dylight 488-conjugated goat anti-rabbit IgG (1:200, Abbkine) and AlexaFluor 594-conjugated donkey anti-goat IgG (1:200, Abcam) secondary antibodies for 60 min at 37°C. NeuN immunofluorescence staining was performed by incubating slices with a primary antibody against NeuN (mouse, 1:200, Abcam, Temecula, CA, USA) followed by an AlexaFluor 594-conjugated goat anti-mouse IgG secondary antibody (1:200, Invitrogen, Carlsbad, CA, USA). Astrocyte and microglial responses were evaluated by immunofluorescence with a GFAP antibody followed by an AlexaFluor 488-conjugated donkey anti-goat IgG (1:200, Abcam) secondary antibody, and immunofluorescence with an Iba1 antibody followed by an AlexaFluor 594-conjugated donkey anti-goat IgG secondary antibody. The nuclei were stained with DAPI (Solarbio Life Sciences, Beijing, China) for 10 min. After mounting, immunofluorescence images were observed with a confocal microscope (Zeiss LSM 880, Carl Zeiss, Germany).

### Statistical Analysis

All data were statistically analyzed using SPSS 22.0 (IBM, Armonk, NY, USA). All graphics were generated with GraphPad Prism 7.0 (GraphPad, La Jolla, CA, USA). The data are expressed as the mean ± SEM unless otherwise indicated. Statistical significance was evaluated by Student’s *t*-test for comparisons between two groups and one-way ANOVA for comparisons between multiple groups. Two-factor (genotype vs. treatment) ANOVA was conducted for comparisons between WT and IDO1^−/−^ groups. When significant interactions were observed, Tukey’s test was used for *post hoc* analysis. The Kruskal–Wallis test was used to compare IDO1 levels and the KYN/TRP ratio between clinical samples. The chi-square test was performed to determine the significance of differences in the severity of SRSs. The Mann–Whitney *U*-test was used to compare the frequency of SRSs between KO epileptic mice and WT epileptic mice. A value of *P* < 0.05 was considered statistically significant.

## Results

### IDO Levels and the KYN/TRP Ratio Were Elevated in the Sera and CSF of Patients With Epilepsy

To investigate the response of IDO1 to epilepsy, we first measured IDO levels and the KYN/TRP ratio in the sera and CSF of patients with epilepsy and controls. The background characteristics of all patients and controls are shown in Table 1. There were no significant differences in age or sex between patients with epilepsy and controls.

**Table 1:** The background characteristics of the patients with epilepsy and controls.

	Epilepsy	Control
Total	21	13
Age (years)	33.05 ± 20.95	34.23 ± 17.26
Gender (female/male)	13/8	7/6
Course of epilepsy		
<1 year	13	0
1–2 years	4	0
>2 years	4	0
Taking antiepileptic drugs	17	0
MRI		
Normal	6	0
Abnormal	13	0
Not available	2	0
Etiology		
Primary epilepsy	9	0
Seizures secondary to autoimmune encephalitis	12	0
SE	11	0
Epileptic patients (without SE)	10	0

**Figure 1:**
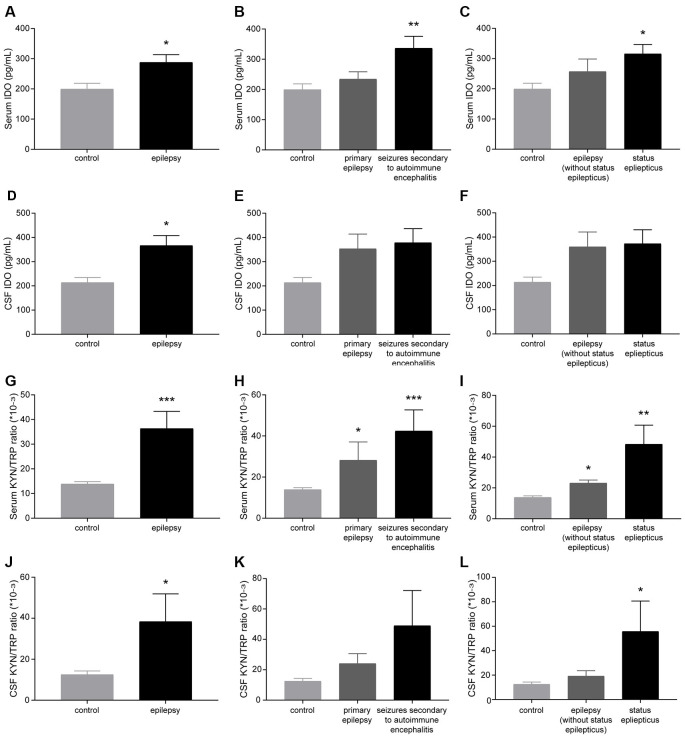
Indoleamine-2,3-dioxygenase (IDO) levels and the kynurenine/tryptophan (KYN/TRP) ratio were elevated in the sera and cerebrospinal fluid (CSF) of patients with epilepsy. **(A–F)** IDO levels in the sera and CSF of participants were evaluated by enzyme-linked immunosorbent assay (ELISA). **(G–L)** The KYN/TRP ratio in the sera and CSF of participants is shown. The data are presented as the mean ± SEM. *P < 0.05, **P < 0.01, ***P < 0.001 compared with controls.

Patients with epilepsy showed a significantly higher IDO level and KYN/TRP ratio (IDO1 activity) in the sera and CSF than controls (Figures 1A,D,G,J). Furthermore, patients with seizures secondary to autoimmune encephalitis had a higher level of IDO in the serum than controls (Figures 1B,E). Patients with SE exhibited a significantly higher IDO level in the serum than controls (Figures 1C,F). Also, compared with controls, both patients with primary epilepsy and patients with seizures secondary to autoimmune encephalitis displayed a progressive increase in IDO1 activity in the serum (Figures 1H,K). IDO1 activity was significantly higher in the sera and CSF of patients with epilepsy compared with controls, especially in patients with SE (Figures 1I,L). Collectively, these results indicated that IDO1 might be associated with human epilepsy.

### IDO1 Levels and the KYN/TRP Ratio Were Elevated in the Sera and Hippocampi of Epileptic Mice

**Figure 2:**
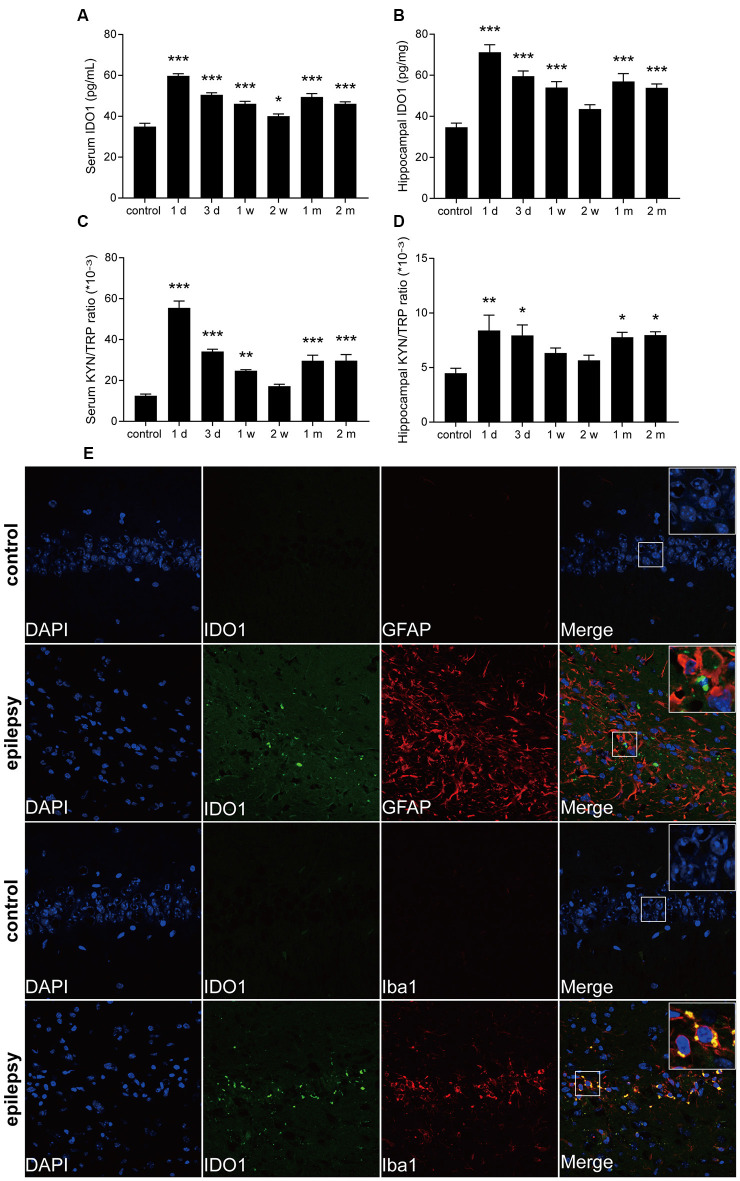
IDO1 levels and the KYN/TRP ratio were increased in the sera and hippocampi of epileptic mice. **(A,B)** IDO1 levels in the sera and hippocampi of epileptic mice were evaluated by ELISA. **(C,D)** The KYN/TRP ratio in the sera and hippocampi of epileptic mice is shown. **(E)** Double immunofluorescence showing colocalization between IDO1 and GFAP or Iba1 in the hippocampi of epileptic mice (magnification: 400×). The data are presented as the mean ± SEM, *n* = 3–6 per group. *P < 0.05, **P < 0.01, ***P < 0.001 compared with the control group.

To further confirm that IDO1 levels are altered in epileptic mice, we established a lithium-pilocarpine-induced epilepsy model and assessed IDO1 levels and the KYN/TRP ratio in the serum and hippocampus at different time points. Ninety percent of the lithium-pilocarpine-injected mice developed SE corresponding to stage 4 or 5 seizures; 92.6% of these animals survived after SE and were used for further experimentation. IDO1 levels and the KYN/TRP ratio were significantly increased in the serum and hippocampus during the acute period (1 day and 3 days) and chronic phase (1 month and 2 month). Notably, IDO1 levels and the KYN/TRP ratio peaked 1 day after SE (Figures 2A–D). These findings verified that there was a strong relationship between the upregulation of IDO1 and epilepsy in these mice.

Then, immunofluorescence was used to assess the distribution of IDO1 in the hippocampi of epileptic mice. The results showed that almost no IDO1 immunoreactivity was observed in the hippocampi of control mice and that IDO1 immunoreactivity was clearly observed in the hippocampi of epileptic mice. IDO1 was mainly observed in the cytosol and perinuclear region in the hippocampus. IDO1 (green) and Iba1 (red) were co-expressed in microglial cells (Figure 2E). The results indicated that microglia-induced IDO1 activation was prominent in epileptic mice.

### Pro-inflammatory Cytokines Levels Were Increased in the Sera and Hippocampi of Epileptic Mice

**Figure 3:**
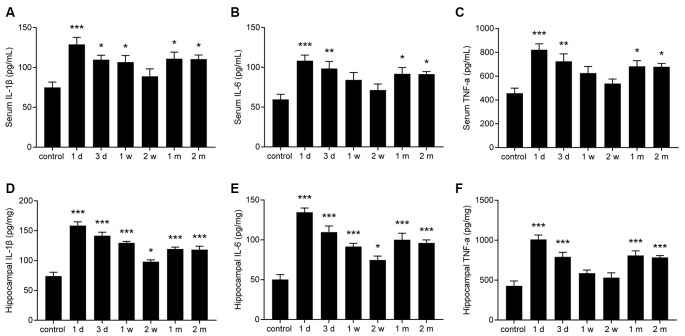
The levels of interleukin (IL)-1β, IL-6, tumor necrosis factor-α (TNF-α) were increased in the sera and hippocampi of epileptic mice. **(A–F)** Bar graphs showing the levels of IL-1β **(A,D)**, IL-6 **(B,E)**, and TNF-α **(C,F**) in the sera and hippocampi of epileptic mice. The data are presented as the mean ± SEM, *n* = 6 per group. *P < 0.05, **P < 0.01, ***P < 0.001 compared with the control group.

To evaluate the change in pro-inflammatory cytokines in epileptic mice, the levels of IL-1β, IL-6, and TNF-α in the sera and hippocampi of mice were measured at different time points. The levels of IL-1β, IL-6, and TNF-α were significantly increased in the serum and hippocampus during the acute period and chronic phase (Figures 3A–F).

### IDO1 Deficiency Suppressed Seizures in the Lithium-Pilocarpine-Induced Epilepsy Model

**Figure 4:**
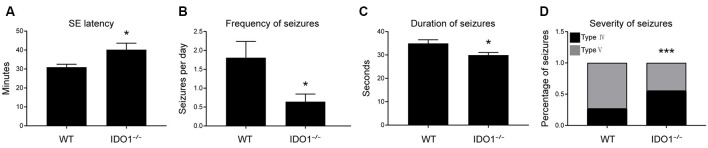
IDO1 deficiency suppressed seizures in a lithium-pilocarpine-induced epilepsy model. **(A)** Latency to status epilepticus (SE) onset after pilocarpine injection in the wild-type (WT) group and IDO^−/−^ group. **(B)** Spontaneous recurrent seizures (SRS) frequency in the WT group and IDO^−/−^ group. **(C)** SRS duration in the WT group and IDO^−/−^ group. **(D)** SRS severity in the WT group and IDO^−/−^ group. The data are presented as the mean ± SEM, *n* = 10–12 per group. *P < 0.05, ***P < 0.001 compared with the WT group.

Next, IDO1^−/−^ mice were used to evaluate the role of IDO1 in epileptic mice. We explored whether IDO1 would affect seizures in the lithium-pilocarpine-induced epilepsy model. Lithium and pilocarpine injections are known to induce SE in mice. Thus, we first sought to assess differences in SE induction between IDO1^−/−^ mice and WT mice. The latency to SE was slightly longer in IDO1^−/−^ mice than in WT mice (Figure 4A). Following SE induction, mice developed chronic SRSs. We also sought to determine whether IDO1 affected SRSs. No seizures were observed in the control group during the experimental period. The frequency of SRSs was lower and the duration of SRSs was shorter in the IDO1-deficient pilocarpine-induced epilepsy group than in the WT pilocarpine-induced epilepsy group (Figures 4B,C). Additionally, the IDO1^−/−^ group exhibited a markedly lower percentage of stage 5 seizures than the WT group (Figure 4D). These results suggested that IDO1 deficiency suppressed seizures in the lithium-pilocarpine-induced epilepsy model.

### IDO1 Deficiency Improved Neuronal Survival in the Lithium-Pilocarpine-Induced Epilepsy Model

**Figure 5:**
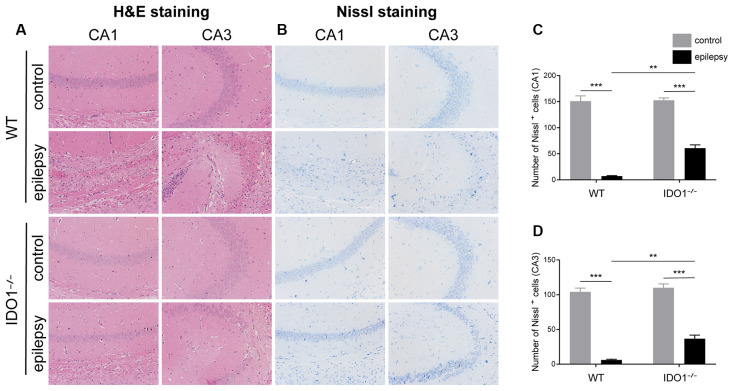
IDO1 deficiency ameliorated neuronal injury.** (A)** Hematoxylin-Eosin (H&E) staining of the hippocampal CA1 and CA3 regions (magnification: 200×). **(B)** Nissl staining of the hippocampal CA1 and CA3 regions (magnification: 200×). **(C,D)** Quantitative analysis of live neurons in the hippocampal CA1 and CA3 regions by Nissl staining. The data are presented as the mean ± SEM, *n* = 3 per group. **P < 0.01, ***P < 0.001.

**Figure 6:**
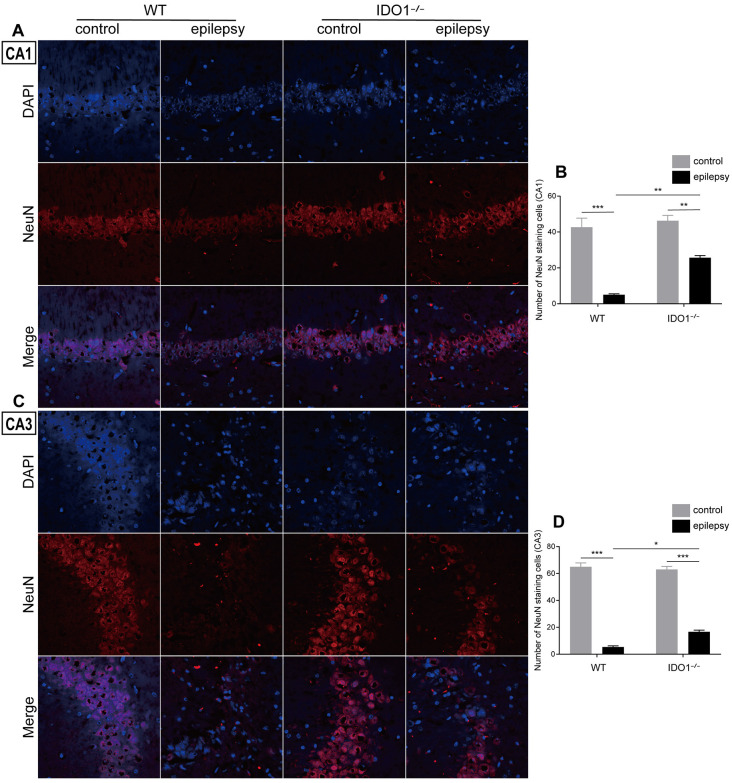
IDO1 deficiency improved hippocampal neuron survival.** (A,C)** Immunofluorescence staining for NeuN in the hippocampal CA1 and CA3 regions (magnification: 400×). **(B,D)** Quantitative analysis of NeuN-positive neurons in the hippocampal CA1 and CA3 regions. The data are presented as the mean ± SEM, *n* = 3 per group. *P < 0.05, **P < 0.01, ***P < 0.001.

Then, we determined whether IDO1 affects neuronal loss in epileptic mice. H&E staining was used to evaluate the pathological changes in the hippocampus in this model. Genetic deletion of IDO1 markedly attenuated the pathological changes in the hippocampal CA1 and CA3 regions in epileptic mice, as assessed by H&E staining (Figure 5A). Nissl staining and NeuN immunostaining were then conducted to further assess the survival of neurons in the hippocampal CA1 and CA3 regions. The results showed that the number of surviving neurons in the hippocampal CA1 and CA3 regions was significantly decreased in the pilocarpine-induced mice (Figures 5B–D, 6A–D, pilocarpine effect in Nissl staining: *F*_(1,8)_ = 337.1, *P* < 0.0001, for CA1; *F*_(1,8)_ = 325.8, *P* < 0.0001, for CA3; pilocarpine effect in NeuN immunostaining: *F*_(1,8)_ = 94.52, *P* < 0.0001, for CA1; *F*_(1,8)_ = 707.2, *P* < 0.0001, for CA3). Among mice with pilocarpine-induced epilepsy, IDO1^−/−^ mice showed significantly more surviving neurons than WT mice (Figures 5B–D, 6A–D, genotype × treatment interaction in Nissl staining: *F*_(1,8)_ = 16.37, *P* = 0.0037, for CA1; *F*_(1,8)_ = 6.752, *P* = 0.0317, for CA3; genotype × treatment interaction in NeuN immunostaining: *F*_(1,8)_ = 8.028, *P* = 0.0220, for CA1; *F*_(1,8)_ = 11.19, *P* = 0.0102, for CA3). Importantly, there were no baseline differences in the survival of neurons in the hippocampus between control groups. These results suggested that IDO1 deficiency partly prevented seizure-induced hippocampal neuronal damage in this model.

### IDO1 Deficiency Reduced the Levels of Neurotoxic KYN Metabolites in the Lithium-Pilocarpine-Induced Epilepsy Model

**Figure 7:**
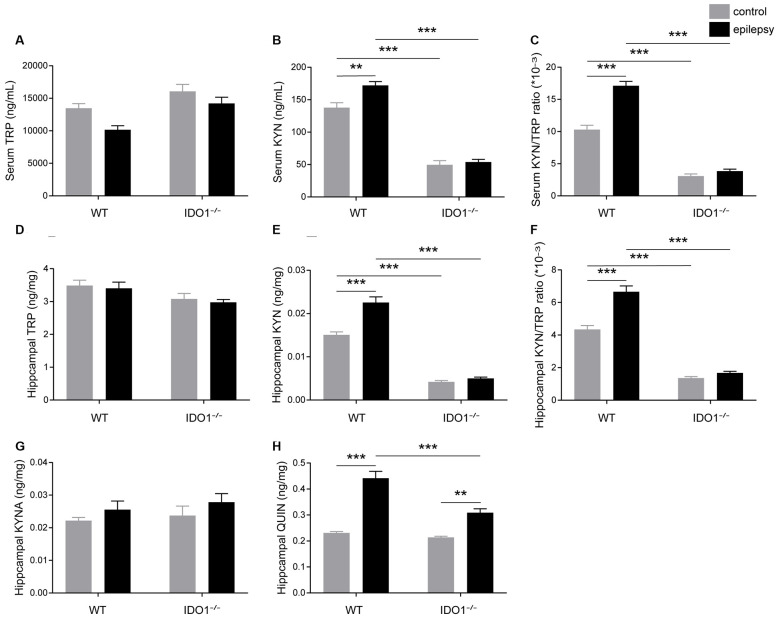
IDO1 deficiency reduced the levels of neurotoxic KYN pathway metabolites. **(A,B,D,E)** LC-MS analysis was used to measure the concentrations of TRP and KYN in the serum and hippocampal tissues of WT and IDO1^−/−^ mice. **(C,F)** The KYN/ TRP ratio in the serum and hippocampus is shown. **(G,H)** LC-MS analysis was used to measure the concentrations of quinolinic acid (QUIN) and kynurenic acid (KYNA) in the hippocampal tissues from WT and KO control and model mice. The data are presented as the mean ± SEM, *n* = 6 per group. **P < 0.01, ***P < 0.001.

We further investigated the impact of IDO1 on the KYN pathway of TRP metabolism. To this end, we first measured the concentrations of TRP and KYN in the sera and hippocampi of WT and IDO1^−/−^ control mice. Pilocarpine challenge increased the concentration of KYN and KYN/TRP ratio in the serum and hippocampus of WT, but not IDO1^−/−^ mice (Figures 7B,E, pilocarpine effect: *F*_(1,20)_ = 10.05, *P* = 0.0048, for serum KYN; *F*_(1,20)_ = 28.12, *P* < 0.0001, for hippocampal KYN; *F*_(1,20)_ = 51.86, *P* < 0.0001, for serum KYN/TRP; *F*_(1,20)_ = 34.54, *P* < 0.0001, for hippocampal KYN/TRP). However, no significant difference was observed between the concentration of TRP in epileptic group and control group (Figures 7A,D). Interestingly, the concentration of KYN in the serum and hippocampus was significantly lower in IDO1^−/−^ control mice than in WT control mice (Figures 7B,E, genotype effect: *F*_(1,20)_ = 284.7, *P* < 0.0001, for serum KYN; *F*_(1,20)_ = 331.9, *P* < 0.0001, for hippocampal KYN). Consistently, the KYN/TRP ratio was significantly lower in IDO1^−/−^ control mice than in WT control mice (Figures 7C,F, genotype effect: *F*_(1,20)_ = 378.7, *P* < 0.0001, for serum KYN/TRP; *F*_(1,20)_ = 317.7, *P* < 0.0001, for hippocampal KYN/TRP). *Post hoc* analysis revealed that the concentration of KYN and KYN/TRP ratio were significantly lower in IDO1^−/−^ epileptic mice than in WT epileptic mice (Figures 7B,C,E,F, genotype × treatment interaction: *F*_(1,20)_ = 6.02, *P* = 0.0234, for serum KYN; *F*_(1,20)_ = 18.29, *P* = 0.0004, for hippocampal KYN; *F*_(1,20)_ = 32.83, *P* < 0.0001, for serum KYN/TRP; *F*_(1,20)_ = 19.99, *P* = 0.0002, for hippocampal KYN/TRP). These results indicated that IDO1 deficiency might reduce the production of KYN and KYN/TRP ratio. We further examined changes in the concentrations of KYNA and QUIN in the hippocampus. No noticeable difference was found in the concentration of KYNA between WT epileptic mice and IDO1^−/−^ epileptic mice (Figure 7G). However, the concentration of QUIN in the hippocampus was significantly increased in the pilocarpine-induced mice (Figure 7H, pilocarpine effect: *F*_(1,20)_ = 97.65, *P* < 0.0001). In addition, QUIN production in the hippocampal tissues from IDO1^−/−^ epileptic mice was less than that in WT epileptic mice (Figure 7H, genotype × treatment interaction: *F*_(1,20)_ = 13.99, *P* = 0.0013). Taken together, IDO1 deficiency reduced neurotoxic KYN metabolite production in epileptic conditions.

### IDO1 Deficiency Ameliorated Inflammatory Responses and Oxidative Stress After SE

**Figure 8:**
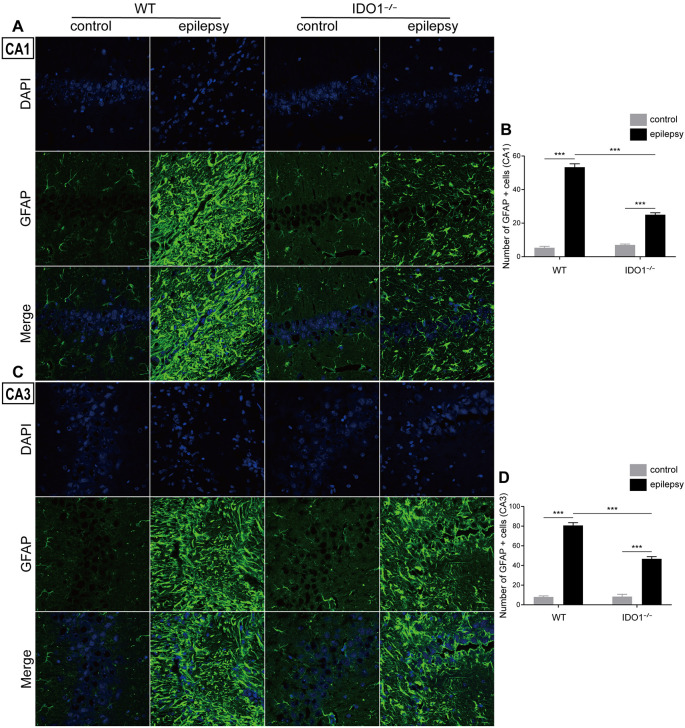
IDO1 deficiency inhibited astrocyte activation in the hippocampus. **(A,C)** Representative images of GFAP immunostaining in the hippocampal CA1 and CA3 regions (magnification: 400×). **(B,D)** Quantification of GFAP^+^ cells in the hippocampal CA1 and CA3 regions. The data are presented as the mean ± SEM, *n* = 3 per group. ***P < 0.001.

**Figure 9:**
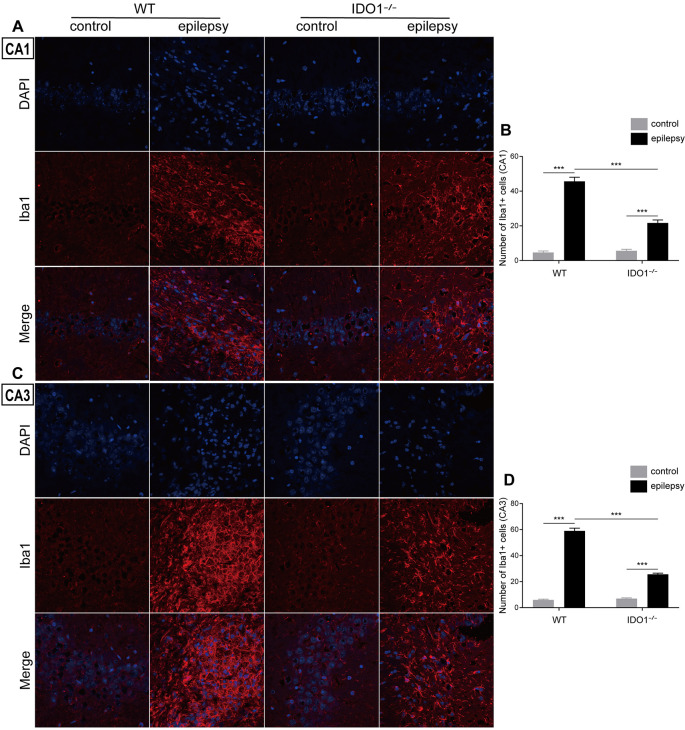
IDO1 deficiency inhibited microglial activation in the hippocampus. **(A,C)** Representative images of Iba1 immunostaining in the hippocampal CA1 and CA3 regions (magnification: 400×). **(B,D)** Quantification of Iba1^+^ cells in the hippocampal CA1 and CA3 regions. The data are presented as the mean ± SEM, *n* = 3 per group. ***P < 0.001.

**Figure 10:**
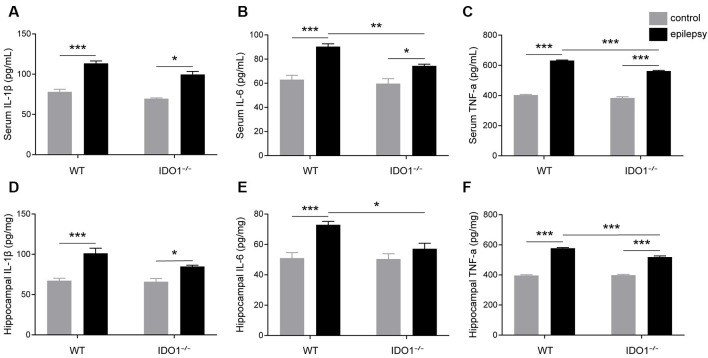
IDO1 deficiency reduced the levels of IL-6 and TNF-α. **(A–F)** Bar graphs showing the levels of IL-1β **(A,D)**, IL-6 **(B,E)**, and TNF-α **(C,F)** in the sera and hippocampi of WT and KO control and model mice. The data are presented as the mean ± SEM, *n* = 6 per group. *P < 0.05, **P < 0.01, ***P < 0.001.

To explore how inflammatory processes are affected by IDO1, glial cell activation and the levels of pro-inflammatory cytokines were analyzed. In the control groups, scattered GFAP-positive astrocytes were distributed in the hippocampal CA1 and CA3 regions. In the epileptic groups, the hippocampal CA1 and CA3 regions were filled with GFAP-positive astrocytes (Figures 8A–D, pilocarpine effect: *F*_(1,8)_ = 664.5, *P* < 0.0001, for CA1; *F*_(1,8)_ = 586.7, *P* < 0.0001, for CA3). IDO1^−/−^ epileptic mice displayed markedly fewer GFAP-positive astrocytes than their WT counterparts (Figures 8B,D, genotype × treatment interaction: *F*_(1,8)_ = 137.3, *P* < 0.0001, for CA1; *F*_(1,8)_ = 56.13, *P* < 0.0001, for CA3). Immunofluorescence staining revealed that the changes in activated microglia in the hippocampi of epileptic mice were consistent with the changes in activated astrocytes (Figures 9A–D, pilocarpine effect: *F*_(1,8)_ = 321.3, *P* < 0.0001, for CA1; *F*_(1,8)_ = 888.9, *P* < 0.0001, for CA3). The number of Ibal-positive microglia was lower in IDO1^−/−^ epileptic mice than in WT epileptic mice (Figures 9B,D, genotype × treatment interaction: *F*_(1,8)_ = 61.81, *P* < 0.0001, for CA1; *F*_(1,8)_ = 204, *P* < 0.0001, for CA3). To investigate the effect of IDO1 deficiency on the pro-inflammatory cytokines, the levels of IL-1β, IL-6 and TNF-α in the serum and hippocampus were determined. ELISA showed that the levels of IL-1β, IL-6 and TNF-α in the sera and hippocampi were significantly increased in the pilocarpine-induced mice (Figures 10A–F, pilocarpine effect: *F*_(1,20)_ = 127.4, *P* < 0.0001, for serum IL-1β; *F*_(1,20)_ = 41.09, *P* < 0.0001, for hippocampal IL-1β; *F*_(1,20)_ = 47.27, *P* < 0.0001, for serum IL-6; *F*_(1,20)_ = 19.03, *P* = 0.0003, for hippocampal IL-6; *F*_(1,20)_ = 1320, *P* < 0.0001, for serum TNF-α; *F*_(1,20)_ = 835.7, *P* < 0.0001, for hippocampal TNF-α). *Post hoc* analysis revealed that the levels of IL-6 and TNF-α in the sera and hippocampi were significantly lower in IDO1^−/−^ epileptic mice than in WT epileptic mice (Figures 10B,C,E,F, genotype × treatment interaction: *F*_(1,20)_ = 4.385, *P* = 0.0492, for serum IL-6; *F*_(1,20)_ = 5.345, *P* = 0.0316, for hippocampal IL-6; *F*_(1,20)_ = 19.3, *P* = 0.0003, for serum TNF-α; *F*_(1,20)_ = 34.38, *P* < 0.0001, for hippocampal TNF-α). These results indicated that genetic IDO1 ablation plays a protective role in epilepsy by modulating inflammatory responses.

**Figure 11:**
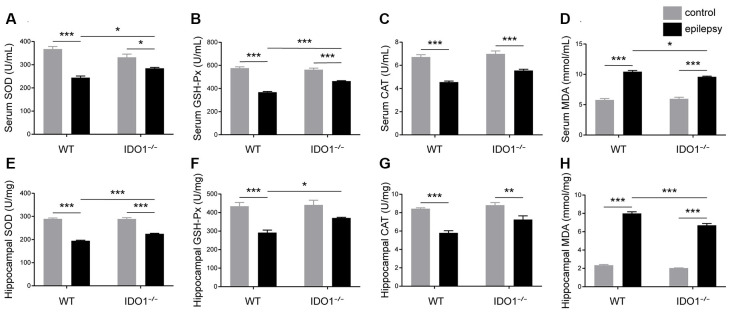
IDO1 deficiency alleviated oxidative stress. **(A–H)** Bar graphs showing the activity of SOD** (A,E)**, GSH-Px **(B,F)**, and CAT **(C,G)** and MDA content **(D,H)** in the sera and hippocampi of WT and KO control and model mice. The data are presented as the mean ± SEM, *n* = 6 per group. *P < 0.05, **P < 0.01, ***P < 0.001.

To assess the effect of IDO1 on oxidative stress, the activity of SOD, GSH-Px and CAT and MDA content in the serum and hippocampus were measured. Our data revealed that pilocarpine challenge decreased SOD, GSH-Px and CAT activity and increased MDA content in the serum and hippocampus (Figures 11A–H, pilocarpine effect: *F*_(1,20)_ = 76.51, *P* < 0.0001, for serum SOD; *F*_(1,20)_ = 410.3, *P* < 0.0001, for hippocampal SOD; *F*_(1,20)_ = 256.1, *P* < 0.0001, for serum GSH-Px; *F*_(1,20)_ = 35.81, *P* < 0.0001, for hippocampal GSH-Px; *F*_(1,20)_ = 101.7, *P* < 0.0001, for serum CAT; *F*_(1,20)_ = 58.41, *P* < 0.0001, for hippocampal CAT; *F*_(1,20)_ = 406.4, *P* < 0.0001, for serum MDA; *F*_(1,20)_ = 1378, *P* < 0.0001, for hippocampal MDA). Antioxidant enzyme activity in the sera and hippocampi were significantly higher in IDO1^−/−^ epileptic mice than in WT epileptic mice, as indicated by higher levels of SOD and GSH-Px (Figures 11A,B,E,F, genotype × treatment interaction: *F*_(1,20)_ = 15.21, *P* = 0.0009, for serum SOD; *F*_(1,20)_ = 14.53, *P* = 0.0011, for hippocampal SOD; *F*_(1,20)_ = 32.33, *P* < 0.0001, for serum GSH-Px; *F*_(1,20)_ = 4.633, *P* = 0.0438, for hippocampal GSH-Px). Correspondingly, MDA content in the serum and hippocampus was significantly lower in IDO1^−/−^ epileptic mice than in WT epileptic mice (Figures 11D,H, genotype × treatment interaction: *F*_(1,20)_ = 6.276, *P* = 0.0210, for serum MDA; *F*_(1,20)_ = 12.68, *P* = 0.0020, for hippocampal MDA). Our results indicated that IDO1 deficiency exerts a protective effect against seizure-induced oxidative stress.

## Discussion

In the current study, we found that IDO levels and the KYN/TRP ratio were increased in the sera and CSF of patients with epilepsy. Additionally, IDO1 levels and the KYN/TRP ratio were increased in the sera and hippocampi of animal models. IDO1 was localized in microglia in epileptic mice. Deletion of IDO1 prolonged the latency to SE and alleviated the severity of SRSs. We further demonstrated that IDO1 deficiency increased neuron survival and decreased the levels of neurotoxic KYN metabolites, glial cell activation, pro-inflammatory cytokine production, and oxidative stress.

IDO1 is known to be the initial and rate-limiting enzyme in the KYN pathway of TRP metabolism. Recent studies have proposed that IDO1 is involved in neurological diseases. Published literature has shown that IDO1 activity is increased in the blood of Huntington’s disease patients (Veres et al., 2015). It was reported that IDO1 levels are increased in the brain in the context of Alzheimer’s disease (Guillemin et al., 2005; Wu et al., 2013). As shown in a previous study, IDO1 contributes to epilepsy-associated depression-like behavior in chronic temporal lobe epilepsy. 1-Methyltryptophan attenuates depressive-like behavior but not spontaneous seizures (Xie et al., 2014). However, recent research indicates that IDO1 deletion increases the incidence of seizures in acute TMEV-induced encephalitis (Juda et al., 2019). Here, we observed that IDO levels and the KYN/TRP ratio were increased in the sera and CSF of patients with epilepsy. Notably, compared with controls, both patients with primary epilepsy and patients with seizures secondary to autoimmune encephalitis displayed a higher serum KYN/TRP ratio.

We further evaluated the changes in IDO1 levels and the KYN/TRP ratio using the lithium-pilocarpine-induced animal model, which is a classic model of epilepsy. In this model, epilepsy can be divided into the acute phase, latent phase, and chronic phase. Pilocarpine-treated mice exhibit SE and repetitive limbic seizures in the acute phase. Then, mice enter the seizure-free phase and display normal behavior. The latent phase varies from 3 days to 2 week after SE. Finally, mice progress into the chronic phase, which is characterized by the occurrence of SRSs (Curia et al., 2008). These changes are analogous to the characteristics of human temporal lobe epilepsy (Zeng et al., 2018). The IDO1 levels and KYN/TRP ratio in the serum and hippocampus were significantly increased during the acute and chronic phases. These changes may have been due to seizures, which were observed during the acute and chronic phases. Also, SE led to a rapid elevation of IDO1 levels and the KYN/TRP ratio in the serum and hippocampus in the animal model. Patients with SE displayed a marked elevation of IDO1 levels and the KYN/TRP ratio in the serum. SE, one of the most common neurologic emergencies, is a serious form of seizure (Betjemann and Lowenstein, 2015). Although we were unable to compare hippocampal IDO1 levels and the KYN/TRP ratio between patients with epilepsy and controls due to practical and ethical limitations, these findings provide evidence that IDO1 levels and the KYN/TRP ratio might be associated with the severity of behavioral seizures.

Under normal conditions, IDO1 is not expressed in many cells. Indeed, IDO1 expression and activity are induced in response to inflammatory processes (Heisler and O’Connor, 2015). IDO1 levels are elevated in a variety of cells, including macrophages, dendritic cells, epithelial cells, microglia, and astrocytes (Fujigaki et al., 2017). In the central nervous system, IDO1 catalyzes the production of different KYN metabolites in microglia and astrocytes due to differences in the types of enzymes expressed. QUIN is only produced in microglia, while KYNA is mostly produced in astrocytes (Lim et al., 2017). To study the role of IDO1, we further assessed the localization of IDO1 in epileptic brain tissue. The results showed that IDO1 was expressed in microglia in epileptic mice.

In this study, IDO1 levels and IDO1 activity were increased in both patients with epilepsy and epileptic mice. IDO1 is a cytokine-inducible rate-limiting enzyme. It has been reported that high levels of pro-inflammatory cytokines (e.g., IL-1β, TNF-α, and IL-6) can be detected in epileptogenic tissue from patients and animal models (Aronica and Crino, 2011; Vezzani et al., 2011). Inflammatory cytokines can induce IDO1 activation. In line with this finding, we found that IL-1β, IL-6, and TNF-α levels were increased in the serum and hippocampus during the acute and chronic phases. These pro-inflammatory cytokines may be involved in activating IDO1 in epileptic mice.

To further explore whether IDO1 could affect epilepsy, we established a lithium-pilocarpine-induced epilepsy model in IDO1^−/−^ mice and WT mice. We found that IDO1 deficiency prolonged the latency to SE and attenuated the severity of SRSs. A previous study showed that IDO1 deletion promotes TMEV-induced seizures (Juda et al., 2019). Conversely, our study revealed that IDO1 deficiency suppressed seizures in the lithium-pilocarpine model of epilepsy. This difference may have been the result of the different models used in the studies. In our research, pilocarpine was used to induce epilepsy. Notably, pilocarpine administration can reproduce the typical pathological changes, and SRSs observed in patients with epilepsy. Thus, pilocarpine is widely employed in basic epilepsy research. Therefore, our results suggest that IDO1 might be involved in the development of epilepsy.

Neuronal loss is one of the typical pathologic hallmarks of temporal lobe epilepsy (Helmstaedter et al., 2003; Han et al., 2018). Seizures can lead to neuronal damage in the hippocampus. Neuronal loss in the hippocampus can in turn promote the progression of temporal lobe epilepsy (Tome Ada et al., 2010). Our results were consistent with previous reports. Also, IDO1^−/−^ model mice exhibited greater neuronal survival than WT model mice.

IDO1 degrades TRP to KYN and leads to the subsequent production of neuroactive KYN metabolites such as KYNA and 3-HK (Ganong and Cotman, 1986). The 3-HK is finally metabolized to QUIN. QUIN is a metabolite that exerts neurotoxic effects by acting specifically on NMDA receptors. QUIN can also elicit neurotoxicity by generating reactive oxygen species and activating glial cells (Fujigaki et al., 2017). The 3-HK is also considered a neurotoxic metabolite that induces oxidative stress (Vécsei et al., 2013). KYNA acts as an NMDA receptor antagonist and has a neuroprotective effect against excitotoxicity-induced neuronal death (Vécsei et al., 2013). However, KYNA leads to glial cell activation and the release of inflammatory factors (Fujigaki et al., 2017). Thus, we hypothesized that IDO1 might mediate neurotoxic KYN metabolites to affect the pathogenesis of epilepsy. Our study revealed that the targeted deletion of IDO1 markedly decreased the production of KYN and the KYN/TRP ratio in the serum and hippocampus. Furthermore, IDO1 deficiency decreased the concentration of QUIN in the hippocampi of epileptic mice. Microglia alone are responsible for producing detectable amounts of QUIN in the central nervous system. We found that IDO1 was expressed in microglia in epileptic mice. Therefore, the elevation of IDO1 levels in microglia that produce QUIN might be an important factor in the pathogenesis of epilepsy.

It has been reported that IDO1 has immunoregulatory properties (Yeung et al., 2015). QUIN has a pro-inflammatory effect on glial cells and upregulates the expression of pro-inflammatory chemokines and cytokines (Mechawar and Savitz, 2016). Converging evidence suggests that inflammation plays an essential role in the pathogenesis of epilepsy (Li et al., 2018). Neuroinflammation arises in the brain after SE and is closely associated with SRSs in the chronic period in epileptic mice. Neuroinflammation is characterized by glial cell activation and the production of inflammatory cytokines in epileptic mice (Zhu et al., 2018). Herein, we demonstrated that IDO1 deficiency depletion suppressed astrocyte and microglial activation in the hippocampus of epileptic mice. Also, IDO1 depletion mainly reduced the upregulation of IL-6 and TNF-α in the serum and hippocampus of epileptic mice. These findings suggest that IDO1 deficiency ameliorated SRSs partly by regulating inflammatory responses.

IDO1 is essential for the regulation of oxidative stress (Mo et al., 2020; Zeng et al., 2020). QUIN may promote free radical generation (Perez-De La Cruz et al., 2012). Recent studies have indicated that oxidative stress is a cause of epileptic activity in epilepsy (Ho et al., 2015). During oxidative stress, antioxidant defense systems can scavenge reactive oxygen species through enzymatic antioxidants such as SOD, GSH-Px, and CAT and non-enzymatic antioxidants such as antioxidant vitamins, cofactors, coenzymes, trace elements, and uric acid (Ratnam et al., 2006; Moloney and Cotter, 2018). MDA is generated as one of the end-products of lipid peroxidation and can act as an indicator of lipid peroxidation (Wang et al., 2019; Hu et al., 2020). We revealed that IDO1^−/−^ epileptic mice exhibited reduced MDA content and increased activity of SOD and GSH-Px in the serum and hippocampus. These findings may explain the protective effect of IDO1 deficiency during the development of chronic recurrent seizures.

**Figure 12:**
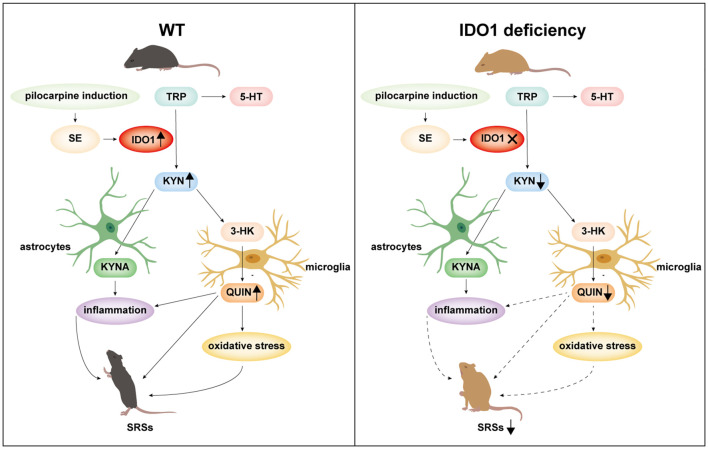
A schematic representation of suppression of seizures by IDO1 deficiency *via* the downregulation of IDO1-dependent neurotoxic metabolites.

In summary, IDO1 is promptly induced in response to epilepsy. IDO1 deficiency suppresses seizures and attenuates neuronal loss in a lithium-pilocarpine-induced epilepsy model. IDO1 deficiency reduces the IDO-dependent production of neurotoxic KYN metabolites, inhibits astrocyte and microglial activation, and decreases the production of pro-inflammatory factors as well as oxidative stress (Figure 12). These results indicate that IDO1 might contribute fundamentally to the progression of epilepsy. Our study provides evidence that IDO1 may be a new therapeutic target for the treatment of temporal lobe epilepsy. However, our results do not provide direct evidence of the underlying mechanism by which IDO1 in microglia regulates the development of epilepsy. More research should be conducted to clarify the detailed mechanisms of how IDO1 affects epilepsy in the future.

## Data Availability Statement

The raw data supporting the conclusions of this article will be made available by the authors, without undue reservation.

## Ethics Statement

The studies involving human participants were reviewed and approved by the Ethics Committee of the Nanfang Hospital, Southern Medical University. Written informed consent to participate in this study was provided by the participants’ legal guardian/next of kin. The animal study was reviewed and approved by the Institutional Animal Care and Use Committee of Southern Medical University.

## Author Contributions

WX designed the experiments. ND, JH, and YH performed the experiments. YD analyzed the data. ND wrote the manuscript. Other authors helped perform the experiments. All authors contributed to the article and approved the submitted version.

## Conflict of Interest

The authors declare that the research was conducted in the absence of any commercial or financial relationships that could be construed as a potential conflict of interest.

## Supplementary Material

Supplementary FIGURE 1 The genotyping results for IDO1^−/−^ mice. The expected size of the IDO1^−/−^ mutant band was 280 bp, while that of the WT band was 427 bp. The primer sequences were as follows: IDO1 oIMR4164: TGG AGC TGC CCG ACG C; IDO1 oIMR4166: TAC CTT CCG AGC CCA GAC AC; IDO1 Oimr6916: CTT GGG TGG AGA GGC TAT TC; and IDO1 Oimr6917: AGG TGA GAT GAC AGG AGA TC.
