# Supplementary figures and images for: Indoleamine-2,3-Dioxygenase 1 Deficiency Suppresses Seizures in Epilepsy
Source: Front Cell Neurosci. 2021 Feb 18;15:638854. doi: 10.3389/fncel.2021.638854 (PMC7935521; doi:10.3389/fncel.2021.638854)

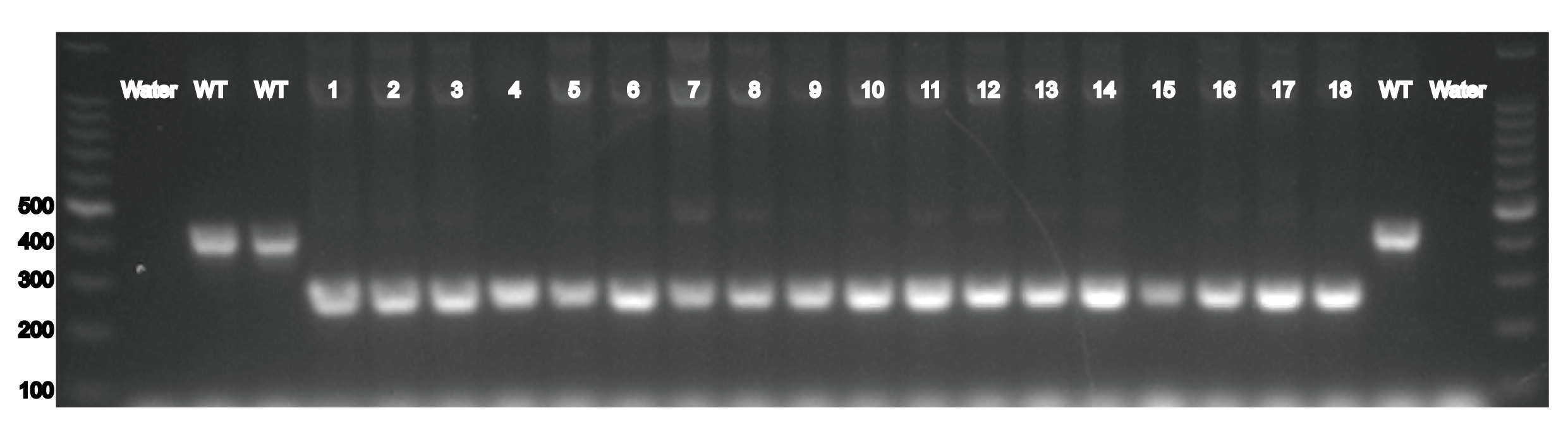

Supplement: Supplementary FIGURE 1 — The genotyping results for IDO1−/− mice. The expected size of the IDO1−/− mutant band was 280 bp, while that of the WT band was 427 bp. The primer sequences were as follows: IDO1 oIMR4164: TGG AGC TGC CCG ACG C; IDO1 oIMR4166: TAC CTT CCG AGC CCA GAC AC; IDO1 Oimr6916: CTT GGG TGG AGA GGC TAT TC; and IDO1 Oimr6917: AGG TGA GAT GAC AGG AGA TC. [file Image_1.TIF]
